# Supplementary material for: Knowledge, attitude, and practice related to the COVID-19 pandemic among undergraduate medical students in Indonesia: A nationwide cross-sectional study
Source: PLoS One. 2022 Jan 21;17(1):e0262827. doi: 10.1371/journal.pone.0262827 (PMC8782366; doi:10.1371/journal.pone.0262827)
Supplement: S1 Table — (DOCX) [file pone.0262827.s001.docx]

**S1 Table.** Characteristics of the study population (n=4870)^a^

| **Variable** | **N (%)** | **Knowledge^b^** | | **Attitude^b^** | | **Practice^b^** | |
| --- | --- | --- | --- | --- | --- | --- | --- |
|  |  | **Inadequate; 3418 (70.2)** | **Adequate; 1452 (29.8)** | **Inadequate; 1710 (35.1)** | **Adequate; 3160 (64.9)** | **Inadequate; 2360 (48.5)** | **Adequate; 2510 (51.5)** |
| Age (years) | 20 (19-21) | 20 (19-21) | 20 (19-22) | 20 (19-21) | 20 (19-21) | 20 (19-21) | 20 (19-21) |
| Sex |  |  |  |  |  |  |  |
| Male | 1471 (30.2) | 1069 (72.7) | 402 (27.3) | 506 (34.4) | 965 (65.6) | 812 (55.2) | 659 (44.8) |
| Female | 3399 (69.8) | 2349 (69.1) | 1050 (30.9) | 1204 (35.4) | 2195 (64.6) | 1548 (45.5) | 1851 (54.5) |
| Location | N=4831 |  |  |  |  |  |  |
| Java | 4199 (86.9) | 2937 (69.9) | 1262 (30.1) | 1465 (34.9) | 2734 (65.1) | 2034 (48.4) | 2165 (51.6) |
| Sumatra | 237 (4.9) | 173 (73.0) | 64 (27.0) | 83 (35.0) | 154 (65.0) | 111 (46.8) | 126 (53.2) |
| Central Indonesia^c^ | 103 (2.1) | 63 (61.2) | 40 (38.8) | 36 (35.0) | 67 (65.0) | 56 (54.4) | 47 (45.6) |
| Eastern Indonesia^d^ | 292 (6.0) | 212 (72.6) | 80 (27.4) | 114 (39.0) | 178 (61.0) | 144 (49.3) | 148 (50.7) |
| Institution type | N=4831 |  |  |  |  |  |  |
| Public | 2525 (52.3) | 1705 (67.5) | 820 (32.5) | 859 (34.0) | 1666 (66.0) | 1325 (52.5) | 1200 (47.5) |
| Private | 2306 (47.7) | 1680 (72.9) | 626 (27.1) | 839 (36.4) | 1467 (63.6) | 1020 (44.2) | 1286 (55.8) |
| Academic level |  |  |  |  |  |  |  |
| Pre-clinical | 3925 (80.6) | 2878 (73.3) | 1047 (26.7) | 1404 (35.8) | 2521 (64.2) | 1928 (49.1) | 1997 (50.9) |
| Clinical | 945 (19.4) | 540 (57.1) | 405 (42.9) | 306 (32.4) | 639 (67.6) | 432 (45.7) | 513 (54.3) |
| Living with^e^ |  |  |  |  |  |  |  |
| Family | 4154 (85.3) | 2930 (70.5) | 1224 (29.5) | 1448 (34.9) | 2706 (65.1) | 2020 (48.6) | 2134 (51.4) |
| Non-family | 58 (1.2) | 40 (69.0) | 18 (31.0) | 20 (34.5) | 38 (65.5) | 32 (55.2) | 26 (44.8) |
| Alone | 658 (13.5) | 448 (68.1) | 210 (31.9) | 242 (36.8) | 416 (63.2) | 308 (46.8) | 350 (53.2) |
| Number of housemate (people)^e^ | 4 (3-5) | 4 (3-5) | 4 (3-5) | 4 (3-5) | 4 (3-5) | 4 (3-5) | 4 (3-5) |
| Living with children |  |  |  |  |  |  |  |
| Yes | 2282 (46.9) | 1615 (70.8) | 667 (29.2) | 792 (34.7) | 1490 (65.3) | 1103 (48.3) | 1179 (51.7) |
| No | 2588 (53.1) | 1803 (69.7) | 785 (30.3) | 918 (35.5) | 1670 (64.5) | 1257 (48.6) | 1331 (51.4) |
| Living with elderly |  |  |  |  |  |  |  |
| Yes | 1155 (23.7) | 786 (68.1) | 369 (31.9) | 394 (34.1) | 761 (65.9) | 563 (48.7) | 592 (51.3) |
| No | 3715 (76.3) | 2632 (70.8) | 1083 (29.2) | 1316 (35.4) | 2399 (64.6) | 1797 (48.4) | 1918 (51.6) |
| Marital status |  |  |  |  |  |  |  |
| Married | 21 (0.4) | 15 (71.4) | 6 (28.6) | 6 (28.6) | 15 (71.4) | 7 (33.3) | 14 (66.7) |
| Not married | 4847 (99.5) | 3402 (70.2) | 1445 (29.8) | 1704 (35.2) | 3143 (64.8) | 2352 (48.5) | 2495 (51.5) |
| Divorced | 2 (0.0) | 1 (50.0) | 1 (50.0) | 0 (0.0) | 2 (100) | 1 (50.0) | 1 (50.0) |
| Family income |  |  |  |  |  |  |  |
| ≤ IDR 1,500,000 | 241 (4.9) | 168 (69.7) | 73 (30.3) | 82 (34.0) | 159 (66.0) | 116 (48.1) | 125 (51.9) |
| IDR 1,500,001-2,500,000 | 273 (5.6) | 200 (73.3) | 73 (26.7) | 119 (43.6) | 154 (56.4) | 154 (56.4) | 119 (43.6) |
| IDR 2,500,001-3,500,000 | 534 (11.0) | 382 (71.5) | 152 (28.5) | 198 (37.1) | 336 (62.9) | 259 (48.5) | 275 (51.5) |
| > IDR 3,500,000 | 3822 (78.5) | 2668 (69.8) | 1154 (30.2) | 1311 (34.3) | 2511 (65.7) | 1831 (47.9) | 1991 (52.1) |
| History of chronic illness |  |  |  |  |  |  |  |
| Yes | 316 (6.5) | 200 (63.3) | 116 (36.7) | 108 (34.2) | 208 (65.8) | 158 (50.0) | 159 (50.0) |
| No | 4554 (93.5) | 3218 (70.7)_ | 1336 (29.3) | 1602 (35.2) | 2952 (64.8) | 2202 (48.4) | 2352 (51.6) |
| Volunteered in health sectors |  |  |  |  |  |  |  |
| Yes | 864 (17.7) | 533 (61.7) | 331 (38.3) | 258 (29.9) | 606 (70.1) | 373 (43.2) | 491 (56.8) |
| No | 4006 (82.3) | 2885 (72.0) | 1121 (28.0) | 1452 (36.2) | 2554 (63.8) | 1987 (49.6) | 2019 (50.4) |
| Volunteered in non-health sectors |  |  |  |  |  |  |  |
| Yes | 3149 (64.7) | 2129 (67.6) | 1020 (32.4) | 1042 (33.1) | 2107 (66.9) | 1473 (46.8) | 1676 (53.2) |
| No | 1721 (35.3) | 1289 (74.9) | 432 (25.1) | 668 (38.8) | 1053 (61.2) | 887 (51.5) | 834 (48.5) |
| Family members diagnosed with COVID-19 |  |  |  |  |  |  |  |
| Yes | 387 (7.9) | 258 (66.7) | 129 (33.3) | 99 (25.6) | 288 (74.4) | 177 (45.7) | 210 (54.3) |
| No | 3831 (78.7) | 2679 (69.9) | 1152 (30.1) | 1378 (36.0) | 2453 (64.0) | 1828 (47.7) | 2003 (52.3) |
| Don’t know | 652 (13.4) | 481 (73.8) | 171 (26.2) | 233 (35.7) | 419 (64.3) | 355 (54.4) | 297 (45.6) |
| Contacts with COVID-19 patients |  |  |  |  |  |  |  |
| Yes | 167 (3.4) | 97 (58.1) | 70 (41.9) | 42 (25.1) | 125 (74.9) | 66 (39.5) | 101 (60.5) |
| No | 3618 (74.3) | 2532 (70.0) | 1086 (30.0) | 1289 (35.6) | 2329 (64.4) | 1694 (46.8) | 1924 (53.2) |
| Don’t know | 1085 (22.3) | 789 (72.7) | 296 (27.3) | 379 (34.9) | 706 (65.1) | 600 (55.3) | 485 (44.7) |
| Had been a COVID-19 patient |  |  |  |  |  |  |  |
| Yes^f^ | 33 (0.7) | 22 (66.7) | 11 (33.3) | 12 (36.4) | 21 (63.6) | 11 (33.3) | 22 (66.7) |
| No | 3990 (81.9) | 2784 (69.8) | 1206 (30.2) | 1423 (35.7) | 2567 (64.3) | 1869 (46.8) | 2121 (53.2) |
| Don’t know | 847 (17.4) | 612 (72.3) | 235 (27.7) | 275 (32.5) | 572 (67.5) | 480 (56.7) | 367 (43.3) |

^a^Unless explicitly stated, data are presented in n (%), mean ± standard deviation, or median (interquartile range). ^b^Knowledge, attitude, and practice was categorized according to the Bloom’s cut-off (≥80%) where a score of ≥8 indicated adequacy of knowledge, and a score of ≥48 indicated adequacy of attitude and practice. ^c^Includes Sulawesi and Kalimantan. ^d^Includes Bali, Nusa Tenggara, Maluku, and Papua. ^e^Defined as the people living with the respondents at the time of questionnaire completion. ^f^Includes both confirmed and unconfirmed (suspected or probable) cases. COVID-19, coronavirus disease 2019; IDR, Indonesian Rupiah.
