# Supplementary material for: Knowledge, attitude, and practice related to the COVID-19 pandemic among undergraduate medical students in Indonesia: A nationwide cross-sectional study
Source: PLoS One. 2022 Jan 21;17(1):e0262827. doi: 10.1371/journal.pone.0262827 (PMC8782366; doi:10.1371/journal.pone.0262827)
Supplement: S2 Table — (DOCX) [file pone.0262827.s002.docx]

**S2 Table.** Item-specific responses on the participants’ knowledge on COVID-19 (n=4870)

| **Signaling question** | **Correct, n (%)** |
| --- | --- |
| 1. Children and teenagers do not need to conduct preventive measures against COVID-19 | 4729 (97.1) |
| 1. SARS-CoV-2, the etiology of COVID-19, is transmitted via respiratory droplets of infected patients | 4697 (96.4) |
| 1. Which of the following can confirm the diagnosis of COVID-19? | 4631 (95.1) |
| 1. The main clinical findings in patients with COVID-19 are fever, fatigue, dry cough, and muscle tenderness (myalgia) | 4087 (83.9) |
| 1. Currently, not a single drug has been proven to cure COVID-19 | 4056 (83.3) |
| 1. In COVID-19 patients, what are the radiological findings that might be seen in the chest CT-scan result? | 3934 (80.8) |
| 1. Cold and nasal congestion are less likely to be found in patients with COVID-19 compared to patients with common cold | 2782 (57.1) |
| 1. What are the laboratory findings that may be present in COVID-19 patients? | 1548 (31.8) |
| 1. Eating or getting into contact with a wild animal might cause someone to get infected by the COVID-19 virus | 1548 (31.8) |
| 1. How long is the median incubation period of SARS-CoV-2 in human body? (in days) | 782 (16.1) |

COVID-19, coronavirus disease 2019; CT-scan, computed tomography scan.
