# Supplementary material for: Knowledge, attitude, and practice related to the COVID-19 pandemic among undergraduate medical students in Indonesia: A nationwide cross-sectional study
Source: PLoS One. 2022 Jan 21;17(1):e0262827. doi: 10.1371/journal.pone.0262827 (PMC8782366; doi:10.1371/journal.pone.0262827)
Supplement: S3 Table — (DOCX) [file pone.0262827.s003.docx]

**S3 Table.** Item-specific responses on the participants’ attitude towards COVID-19 (n=4870)

| **Signaling question** | **Strongly disagree; n (%)** | **Disagree; n (%)** | **Neutral; n (%)** | **Agree; n (%)** | **Strongly agree; n (%)** |
| --- | --- | --- | --- | --- | --- |
| 1. Face mask is effective to prevent COVID-19 infection | 46 (0.9) | 99 (2.0) | 391 (8.0) | 2389 (49.1) | 1945 (39.9) |
| 1. Washing your hands might prevent you from getting COVID-19 infection | 24 (0.5) | 15 (0.3) | 163 (3.3) | 2197 (45.1) | 2471 (50.7) |
| 1. Early detection of COVID-19 might improve the outcomes of treatment | 26 (0.5) | 33 (0.7) | 281 (5.8) | 2205 (45.3) | 2325 (47.7) |
| 1. It is possible to take care of COVID-19 patients in their home | 338 (6.9) | 762 (15.6) | 1636 (33.6) | 1655 (34.0) | 479 (9.8) |
| 1. Health education might reduce the rate of COVID-19 infection | 32 (0.7) | 38 (0.8) | 330 (6.8) | 2165 (44.5) | 2305 (47.3) |
| 1. COVID-19 is a serious disease | 27 (0.6) | 29 (0.6) | 305 (6.3) | 1888 (38.8) | 2621 (53.8) |
| 1. Once a vaccine for COVID-19 is available, I will voluntarily get vaccinated | 29 (0.6) | 42 (0.9) | 703 (14.4) | 1873 (38.5) | 2223 (45.6) |
| 1. COVID-19 is a curable disease | 39 (0.8) | 77 (1.6) | 808 (16.6) | 2362 (48.5) | 1584 (32.5) |
| 1. People living in my surroundings already have a good awareness regarding COVID-19 | 548 (11.3) | 1253 (25.7) | 1503 (30.9) | 1237 (25.4) | 329 (6.8) |
| 1. The government should have prohibited people from traveling to and from areas infected with COVID-19 to prevent transmission | 44 (0.9) | 89 (1.8) | 792 (16.3) | 1985 (40.8) | 1960 (40.2) |
| 1. If the number of COVID-19 cases continues to rise, the government should close public places/areas. (e.g., school, place of worship, shopping center, etc.) | 36 (0.7) | 70 (1.4) | 584 (12.0) | 1850 (38.0) | 2330 (47.8) |
| 1. If the number of COVID-19 cases continues to rise, the government should be ready to impose a lockdown policy | 51 (1.0) | 89 (1.8) | 644 (13.2) | 1726 (35.4) | 2360 (48.5) |

COVID-19, coronavirus disease 2019.
