# Supplementary material for: Knowledge, attitude, and practice related to the COVID-19 pandemic among undergraduate medical students in Indonesia: A nationwide cross-sectional study
Source: PLoS One. 2022 Jan 21;17(1):e0262827. doi: 10.1371/journal.pone.0262827 (PMC8782366; doi:10.1371/journal.pone.0262827)
Supplement: S4 Table — (DOCX) [file pone.0262827.s004.docx]

**S4 Table.** Item-specific responses on the participants’ practice towards COVID-19 (n=4870)

| **Signaling question** | **Strongly disagree; n (%)** | **Disagree; n (%)** | **Neutral; n (%)** | **Agree; n (%)** | **Strongly agree; n (%)** |
| --- | --- | --- | --- | --- | --- |
| 1. In this pandemic, I have always washed my hands for at least 20 seconds | 22 (0.5) | 63 (1.3) | 569 (11.7) | 2087 (42.9) | 2129 (43.7) |
| 1. In this pandemic, I have always tried to avoid touching my eyes, mouth, and mouth with my hand directly | 20 (0.4) | 181 (3.7) | 1163 (23.9) | 2176 (44.7) | 1330 (27.3) |
| 1. In this pandemic, I have always implemented a proper coughing and sneezing etiquette | 10 (0.2) | 24 (0.5) | 232 (4.8) | 1695 (34.8) | 2909 (59.7) |
| 1. In this pandemic, I have always tried to use disinfectants or alike-solutions to clean the surface of my surroundings | 41 (0.8) | 146 (3.0) | 594 (12.2) | 1802 (37.0) | 2287 (47.0) |
| 1. In this pandemic, I have always tried to use disinfectants or alike-solutions to clean my handphone | 146 (3.0) | 388 (8.0) | 1060 (21.8) | 1643 (33.7) | 1633 (33.5) |
| 1. In order to prevent contracting and spreading COVID-19, I avoid going out of my home | 57 (1.2) | 77 (1.6) | 474 (9.7) | 2179 (44.7) | 2083 (42.8) |
| 1. In this pandemic, I have always consumed vitamins to strengthen my immune system | 129 (2.6) | 279 (5.7) | 965 (19.8) | 1816 (37.3) | 1681 (34.5) |
| 1. In the last few days, I have consumed antibiotics or other non-herbal medications to prevent COVID-19 infection | 1927 (39.6) | 1118 (23.0) | 890 (18.3) | 525 (10.8) | 410 (8.4) |
| 1. In this pandemic, I have always put my face mask on before getting into contact with other people | 8 (0.2) | 14 (0.3) | 120 (2.5) | 1296 (26.6) | 3432 (70.5) |
| 1. In this pandemic, I have always avoided handshaking, hugging, or kissing | 17 (0.3) | 50 (1.0) | 322 (6.6) | 1754 (36.0) | 2727 (56.0) |
| 1. In this pandemic, I have always tried to avoid crowds and getting into close contact with people | 12 (0.2) | 20 (0.4) | 280 (5.7) | 2025 (41.6) | 2533 (52.0) |
| 1. In this pandemic, I have consumed herbal products or other traditional medicines (e.g., *jamu*, *temulawak*, and other herbal “drugs” intended to affect my health) | 1449 (29.8) | 990 (20.3) | 1056 (21.7) | 821 (16.9) | 554 (11.4) |

COVID-19, coronavirus disease 2019.
