# Supplementary material for: Knowledge, attitude, and practice related to the COVID-19 pandemic among undergraduate medical students in Indonesia: A nationwide cross-sectional study
Source: PLoS One. 2022 Jan 21;17(1):e0262827. doi: 10.1371/journal.pone.0262827 (PMC8782366; doi:10.1371/journal.pone.0262827)
Supplement: S5 Table — (DOCX) [file pone.0262827.s005.docx]

**S5 Table.** Correlation between the students’ level of trust in health information sources with knowledge, attitude, and practice toward COVID-19 (n=4870)^a^

| **Items** | **Knowledge** | **Attitude** | **Practice** |
| --- | --- | --- | --- |
| Television | -0.001 | -0.005 | -0.007 |
| Newspaper | -0.005 | 0.003 | -0.010 |
| Online news | 0.008 | 0.007 | -0.026 |
| Social media | -0.009 | 0.000 | -0.040* |
| Government statement | 0.019 | -0.001 | 0.013 |
| Health institution statement | 0.012 | -0.004 | -0.002 |
| Expert opinion | 0.020 | -0.007 | -0.020 |

*p<0.01. ^a^Analyzed with Spearman’s correlation test. COVID-19, coronavirus disease 2019.
