# Supplementary material for: Knowledge, attitude, and practice related to the COVID-19 pandemic among undergraduate medical students in Indonesia: A nationwide cross-sectional study
Source: PLoS One. 2022 Jan 21;17(1):e0262827. doi: 10.1371/journal.pone.0262827 (PMC8782366; doi:10.1371/journal.pone.0262827)
Supplement: Attachment — Submitted filename: Response to reviewer.docx [file pone.0262827.s006.docx]

December 12^th^, 2021

Dear Editor-in-chief and respected reviewers,

Thank you for the comments from the editor and reviewers on our manuscript entitled **"Knowledge, attitude, and practice related to the COVID-19 pandemic among undergraduate medical students in Indonesia: a nationwide cross-sectional study”** by Imam Adli, Indah S. Widyahening, Gilbert Lazarus, Jason Phowira, Lyanna Azzahra, Bagas Ariffandi, Azis Muhammad Putera, David Nugraha, Nico Gamalliel, Ardi Findyartini (manuscript ID: **PONE-D-21-12734**). We really appreciate the constructive and detailed feedback on our manuscript. We have revised the current submitted manuscript based on the reviewers’ feedback. The texts highlighted in yellow refer to changes made following the reviewers’ suggestions.

**Details of revisions**

| **No** | **Comments and recommendations** | **Revisions** |
| --- | --- | --- |
| **Reviewer 2** | | |
| 1 | To state any pilot study conducted prior to actual data collection. | We have added a statement illustrating the pilot testing which was conducted prior to the actual data collection.  A preliminary survey was then conducted on 30 participants to enhance the comprehension and the validity of the questionnaire [see page 7 line 104-105] |
| 2 | To state any reminder mechanism to increase the number of participants | We have explained the reminder mechanism implemented to maximize the number of respondents in this study  During the data collection period, the questionnaire was spread using social media platforms every three days to increase the number of respondents. [see page 6 line 95-96] |
| 3 | To state any mechanism to avoid duplicates of response (i.e. from same respondent) | We have added a statement explaining the mechanism implemented to avoid duplicate responses:  To avoid duplicate responses, all participants were required to log into their email accounts, but any personal contact information were not recorded to protect the anonymity of the respondents. [see page 6 line 93-95] |
| 4 | To report the overall response rate | Thank you very much for bringing this to our attention. We believe that overall response rate is not applicable in this study as we recruited the participants using a snowball sampling method; where, rather than counting how many times the questionnaire was distributed, we encouraged the participants to distribute the questionnaire as widely as possible. In addition, we were unable to determine the exact number of medical students in Indonesia and hence were unable to assume the total number of questionnaires distributed based on the size of the total population |
| 5 | To state whether or not the data was collected during the lockdown  period in Indonesia. | We have added a sentence specifying that the data was collected during the lockdown periods in Indonesia  Participants were recruited using a snowball sampling technique and all data were collected via an online self-reported questionnaire using Google Forms (http://forms.google.com/) as the data collection period coincided with the implementation of the COVID-19 lockdown policy in Indonesia. [see page 6 line 91-92] |
| 6 | To state any limitation of the study, particularly related to No. 5  above. This is important as there are always new information and  development of CoVID-19 between now and back in end of 2019. | We have added a discussion stating the limitation of our study which may be caused by the temporality of COVID-19 knowledge and evidence.  The generalizability of our findings might also be affected by the constantly evolving evidence on and situation due to COVID-19, thereby implying that the knowledge, attitude, and practice of medical students in Indonesia found in this study may change over time. [see page 21 line 342-344]  During the pandemic, the Indonesian government implemented a community activities restrictions enforcement, which is a continuous restriction period with multiple levels of activity restrictions according to the prevalence of the COVID-19 in the area. Hence, although we agree that the generalizability of our findings may be affected when the lockdown restrictions are lifted, we believe that in the context of COVID-19 in Indonesia, it is more applicable to state that the temporality of COVID-19 knowledge and evidence may exert a greater influence on the generalizability of our findings rather than the lockdown periods. |
| **Reviewer 2** | | |
| 1 | 33: This study aims to assess . . . . | We have revised the statement according to the reviewer’s suggestion  To further explore their potentials as public educators and role models for the communities during the coronavirus disease 2019 (COVID-19) pandemic, this study aims to assess the knowledge, attitude, and practice of these students toward COVID-19. [see page 3 line 30-31] |
| 2 | 36: Socio-demographics  98: Measurement tools  104 and 106: Socio-demographic | We have revised the term accordingly  Socio-demographics characteristics... [see page 3 line 33]  Measurement tools [see page 7 line 100]  (1) socio-demographics characteristics... [see page 7 line 108]  Socio-demographics characteristics consisted of... [see page 7 line 110] |
| 3 | 39: Specify the 64.9% and 51.5% positive attitude and practice | We have specified the percentages for attitude (64.9%) and practice (51.5%) in the abstract  Out of 4870 respondents, 64.9% had positive attitude and 51.5% had positive practice toward COVID-19, while only 29.8% had adequate knowledge [see page 3 line 36-37] |
| 4 | 101: GL, IA, and AMP write full abbreviation | Please note that GL, IA, and AMP refer to the initials of the authors who translated the questionnaire rather than custom abbreviations. To prevent further misunderstanding, we have added an information emphasizing that the text refers to the initials of the authors.  The questionnaire was developed through literature searches of previously validated questionnaires[9–12], which was then translated to Bahasa Indonesia through backward and forward translations by three of the authors (GL, IA and AMP), and modified to match the context of the study [see page 7 line 103] |
| 5 | 122: Explain what do you mean by two 12 five-point Likert, In table it is 5 Likert scale? | Attitude and practice were assessed by using a five-point Likert scale questionnaire consisting of 12 questions each. We have revised the statement to prevent further misinterpretations  In contrast, assessment of attitude and practice consisted of 12 questions each using a five-point Likert scale [see page 8 line 126-127] |
| 6 | 166: What do you mean by older students here? Female and older  students. If possible, specify which age group, how do you interpretate older students. Median age is 20 IQR 19-21. | As the range of age distribution was narrow, we decided to treat the variable as a continuous variable. In this case, older students refer to students with older age, independent of the academic level (pre-clinical or clinical year). We have rephrased the text as following:  Our findings indicated that female (OR 1.22 [95% CI: 1.06-1.40], p=0.006) and students with an older age were more likely to demonstrate a higher level of knowledge (OR 1.06 [95% CI: 1.00-1.12], p=0.034; **Table 1**) [see page 10 line 170-171] |
| 7 | 168: Table 1 is representing the “Odd Ratio (OR)” and need to  rephrase the interpretation in the text, how it has been interpretated  female and older students were more knowledge able | Thank you very much for the suggestion. We have rephrased the interpretation accordingly  Our findings indicated that female (OR 1.22 [95% CI: 1.06-1.40], p=0.006) and older students were more likely to demonstrate a higher level of knowledge (OR 1.06 [95% CI: 1.00-1.12], p=0.034; **Table 1**) [see page 10 line 169-171] |
| 8 | 228: correlation value and p value are different from table S5 | Thank you very much for bringing this to our attention. We have checked the data and revised them accordingly. The correlation coefficient and p-value should be -0.040 and p=0.005, respectively.  Although the negative association between trust in social media and the students’ knowledge was statistically significant, the magnitude of the correlation was clinically negligible (ρ= -0.040; p=0.005**; S5 Table)**. [see page 16 line 232] |
| 9 | Specify the cut off score knowledge, Attitude, and Practice score in Table S1 | We have added a footnote explaining the cut-off scores used to dichotomize the knowledge, attitude, and practice scores in the study.  ^b^Knowledge, attitude, and practice was categorized according to the Bloom’s cut-off (≥80%) where a score of ≥8 indicated adequacy of knowledge, and a score of ≥48 indicated adequacy of attitude and practice [see footnote of Table S1] |
| 10 | Do mention percentage and frequency n (%) in table S3 and S4 | We have added a legend explaining that the values are presented in frequencies and proportions (n (%)) [see Table S3 and S4] |
| 11 | Kindly check the divorced percentage and frequency in Table 2 | There were only 2 respondents which was divorced at the time of data collection (0.04% of the total sample size). As both respondents had a positive attitude (no divorced participants scored <80% in the attitude domain), the model could not converge an odds ratio and 95% confidence interval for the variable. In other words, the available data was insufficient to determine the association between divorced students and attitude towards COVID-19 relative to unmarried students. We have rephrased the text to “Not estimable” to prevent further misunderstanding. [see Table 2] |
| 12 | Concern with your ref value in table 2 and 3. Some independent variables  ref is first variable and in some it is last and some in between... be consistent | We have rearranged the tables by placing the reference category of each variable at the last row. [see Table 1-3] |

We hope you will kindly reconsider our submission and we are looking forward to the publication of our manuscript.

Thank you very much in advance for your kind consideration.

Sincerely yours,

Representing all authors

**Indah Suci Widyahening, MD, M.S., M.Sc-CMFM, PhD**

Department of Community Medicine,

Faculty of Medicine Universitas Indonesia, Jakarta 10430, Indonesia

Phone/Fax No: +62 21 3141066

Email Address: indah_widyahening@ui.ac.id
